# Hyperpolarized [1,4-^13^C_2_]Fumarate Enables Magnetic Resonance-Based Imaging of Myocardial Necrosis

**DOI:** 10.1016/j.jcmg.2017.09.020

**Published:** 2018-11

**Authors:** Jack J. Miller, Angus Z. Lau, Per Mose Nielsen, Giles McMullen-Klein, Andrew J. Lewis, Nichlas Riise Jespersen, Vicky Ball, Ferdia A. Gallagher, Carolyn A. Carr, Christoffer Laustsen, Hans Erik Bøtker, Damian J. Tyler, Marie A. Schroeder

**Affiliations:** a Department of Physiology, Anatomy & Genetics, University of Oxford, Oxford, United Kingdom; b Department of Physics, University of Oxford, Oxford, United Kingdom; c University of Oxford Centre for Clinical Magnetic Resonance Research, Radcliffe Department of Medicine, University of Oxford, Oxford, United Kingdom; d Physical Sciences, Sunnybrook Research Institute, Toronto, Canada; e Department of Clinical Medicine, Aarhus University Hospital Skejby, Aarhus, Denmark; f Department of Radiology, University of Cambridge, Cambridge, United Kingdom

**Keywords:** cardiac MRI, energy metabolism, hyperpolarized MR, magnetic resonance spectroscopy, necrosis, ATP, adenosine triphosphate, CVD, cardiovascular disease, LDH, lactate dehydrogenase, MI, myocardial infarction, MRI, magnetic resonance imaging, mRNA, messenger ribonucleic acid, MRS, magnetic resonance spectroscopy, PCr, phosphocreatine, SNR, signal to noise ratio

## Abstract

**Objectives:**

The aim of this study was to determine if hyperpolarized [1,4–^13^C_2_]malate imaging could measure cardiomyocyte necrosis after myocardial infarction (MI).

**Background:**

MI is defined by an acute burst of cellular necrosis and the subsequent cascade of structural and functional adaptations. Quantifying necrosis in the clinic after MI remains challenging. Magnetic resonance-based detection of the conversion of hyperpolarized [1,4–^13^C_2_]fumarate to [1,4–^13^C_2_]malate, enabled by disrupted cell membrane integrity, has measured cellular necrosis in vivo in other tissue types. Our aim was to determine whether hyperpolarized [1,4–^13^C_2_]malate imaging could measure necrosis after MI.

**Methods:**

Isolated perfused hearts were given hyperpolarized [1,4–^13^C_2_]fumarate at baseline, immediately after 20 min of ischemia, and after 45 min of reperfusion. Magnetic resonance spectroscopy measured conversion into [1,4–^13^C_2_]malate. Left ventricular function and energetics were monitored throughout the protocol, buffer samples were collected and hearts were preserved for further analyses. For in vivo studies, magnetic resonance spectroscopy and a novel spatial-spectral magnetic resonance imaging sequence were implemented to assess cardiomyocyte necrosis in rats, 1 day and 1 week after cryo-induced MI.

**Results:**

In isolated hearts, [1,4–^13^C_2_]malate production became apparent after 45 min of reperfusion, and increased 2.7-fold compared with baseline. Expression of dicarboxylic acid transporter genes were negligible in healthy and reperfused hearts, and lactate dehydrogenase release and infarct size were significantly increased in reperfused hearts. Nonlinear regression revealed that [1,4–^13^C_2_]malate production was induced when adenosine triphosphate was depleted by >50%, below 5.3 mmol/l (R^2^ = 0.904). In vivo, the quantity of [1,4–^13^C_2_]malate visible increased 82-fold over controls 1 day after infarction, maintaining a 31-fold increase 7 days post-infarct. [1,4–^13^C_2_]Malate could be resolved using hyperpolarized magnetic resonance imaging in the infarct region one day after MI; [1,4–^13^C_2_]malate was not visible in control hearts.

**Conclusions:**

Malate production in the infarcted heart appears to provide a specific probe of necrosis acutely after MI, and for at least 1 week afterward. This technique could offer an alternative noninvasive method to measure cellular necrosis in heart disease, and warrants further investigation in patients.

## Graphical abstract

**Figure:**
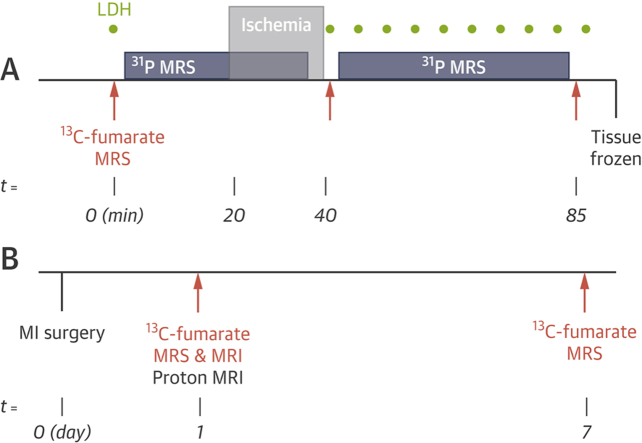

Cell death is a hallmark of cardiovascular disease (CVD), including heart failure and myocardial infarction (MI). Cardiomyocytes die via distinct mechanisms—necrosis, apoptosis, and autophagy—each of which occurs by different signaling events (1). Necrosis is marked by distinct morphologic changes, including cell and organelle swelling, plasma membrane rupture, and depletion of chemical energy in the form of adenosine triphosphate (ATP) 1, 2. Emerging evidence suggests that instead of being a passive form of cell death, necrosis can be regulated by signaling cascades 1, 2. Experimentally, abrogating necrosis improves myocardial outcome 3, 4, 5, 6, 7.

In vivo imaging of necrosis in the clinic remains challenging. Myocardial necrosis associated with ischemia and reperfusion is detected clinically by the presence of cardiomyocyte-specific proteins released into the circulation, ideally troponin. However, circulating troponin assays are not specific to MI, particularly in the settings of renal failure or sepsis (8). Furthermore, biomarkers found in the blood do not enable spatial localization, preventing tissue damage from being assigned to regions of the heart. Imaging-based techniques, including echocardiography, nuclear imaging, and magnetic resonance imaging (MRI) with and without late gadolinium enhancement, are useful in detecting the outcomes associated with necrosis and other cell death, namely, reduced perfusion, local wall motion abnormalities, wall thinning, edema, and scar development 9, 10, 11, 12, 13, 14, 15. Invasive myocardial analysis techniques, including histology, focus on regions of tissue in which cell death causes scar formation 2, 16, 17, 18, 19.

Recently, the metabolic production of [1,4-^13^C_2_]malate from hyperpolarized [1,4-^13^C_2_]fumarate was demonstrated to offer positive MR contrast to identify cellular necrosis in vivo in tumor cells and acute kidney injury 20, 21*.* The fumarate-to-malate hydration reaction is catalyzed by the intracellular enzyme fumarase as part of the tricarboxylic acid cycle. Unlike many metabolic reactions requiring cofactors such as nicotinamide adenine dinucleotide to proceed, the fumarase reaction requires no cofactors and maintains activity during cell death (21). Imaging malate using ^13^C MRI ensured specificity to necrosis by targeting loss of cell membrane integrity: [1,4-^13^C_2_]malate production was only observed when the cell membrane was disrupted, enabling the infused hyperpolarized [1,4-^13^C_2_]fumarate to access the fumarase enzyme (21).

Malate imaging may be valuable to detect necrosis in heart disease. A dicarboxylic acid transporter with a known capacity to import fumarate into cells has not been detected in the heart 22, 23, implying that malate production may only be observed due to cell membrane rupture. Furthermore, the clinical translation of hyperpolarized [1,4-^13^C_2_]fumarate is actively underway and hyperpolarized [1-^13^C]pyruvate is already being used in humans 24, 25. Noninvasive [1,4-^13^C_2_]malate imaging should soon be available for patients.

This study tested the hypothesis that MRI of [1,4-^13^C_2_]malate production could assess the acute burst of cardiomyocyte necrosis characteristic of a MI. Initially, we used the isolated perfused heart to achieve this aim, to confirm the specificity of [1,4-^13^C_2_]malate detection to cell membrane rupture. We then translated the hyperpolarized [1,4-^13^C_2_]fumarate method in vivo, assessing myocardial [1,4-^13^C_2_]malate production at multiple time points after infarction and developing a novel MRI pulse sequence to image [1,4-^13^C_2_]malate with high sensitivity. Our results demonstrate the potential of hyperpolarized [1,4-^13^C_2_]malate imaging to enhance our understanding of the mechanisms driving myocardial necrosis, and to image areas of necrosing myocardium.

## Methods

**Figure 1:**
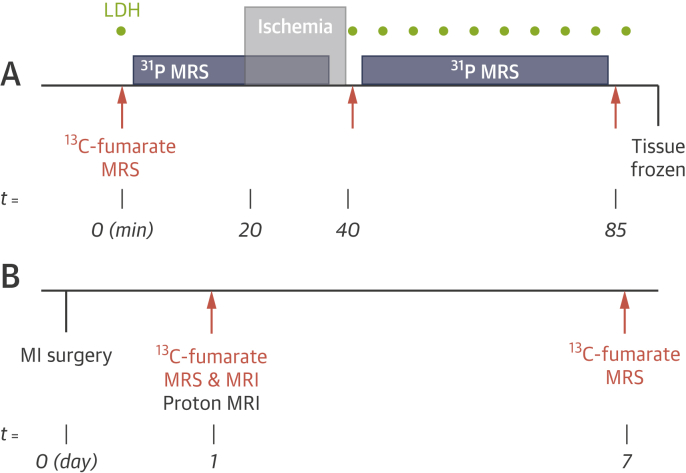
Overview of the Experimental Protocol **(A)** Protocol for the isolated perfused heart experiments. After a stabilization period, hearts were assessed with carbon-13 **(orange)** and phosphorus-31 **(blue)** MRS at baseline, immediately after a 20-min period of total global ischemia, and after 45 min of reperfusion. Buffer samples were collected for LDH measurements **(green)**. After the final carbon-13 scan, hearts were freeze-clamped for subsequent messenger RNA analyses. **(B)** In vivo experimental protocol. Carbon-13 MRS and MRI experiments were performed 1 day after cryo-induction of MI. MRS experiments were repeated after 7 days. LDH = lactate dehydrogenase; MI = myocardial infarction; MRI = magnetic resonance imaging; MRS = magnetic resonance spectroscopy.

All experiments were performed in accordance with relevant UK/Danish legislation and were subject to local ethical review. An overview of the experimental protocol is provided in Figure 1. Further experimental details are available in the Online Appendix.

### Contrast agent preparation

[1,4-^13^C_2_]Fumaric acid was prepared and hyperpolarized as described previously (21). [1,4-^13^C_2_]Fumarate was diluted in the perfusion buffer to 1 mmol/l for perfused heart experiments, and 2 ml of 20 mmol/l [1,4-^13^C_2_]fumarate was infused in vivo over 10 s.

### Perfused heart experiments

Hearts from male Wistar rats (∼300 g; n = 6) were perfused in the Langendorff mode as described previously (26) and as detailed in the Online Appendix. Carbon-13 and phosphorus-31 MR spectra were acquired alternately throughout the protocol. Buffer samples were collected to measure lactate dehydrogenase (LDH) release from necrosing cells, and hearts were freeze-clamped and stored at −80°C for messenger ribonucleic acid (mRNA) analysis. An additional cohort of 7 hearts were reperfused to enable quantification of infarct area due to necrosis histologically using 2,3,5-triphhenyltetrazoliumchloride 27, 28 (Online Appendix).

### In vivo magnetic resonance

Six female Wistar rats (mass ≈200 g; Envigo, Huntingdon, United Kingdom) were divided into 2 groups (cryo-induced MI or control; n = 3 each). Animal handling was performed as described in the Online Appendix.

#### Carbon-13 magnetic resonance spectroscopy

Experiments were performed as described in the Online Appendix, using a 72-mm dual-tuned ^1^H/^13^C proton/carbon birdcage volume coil (Rapid Biomedical GMBH, Rimpar, Germany) (29).

#### Carbon-13 MRI

**Figure 2:**
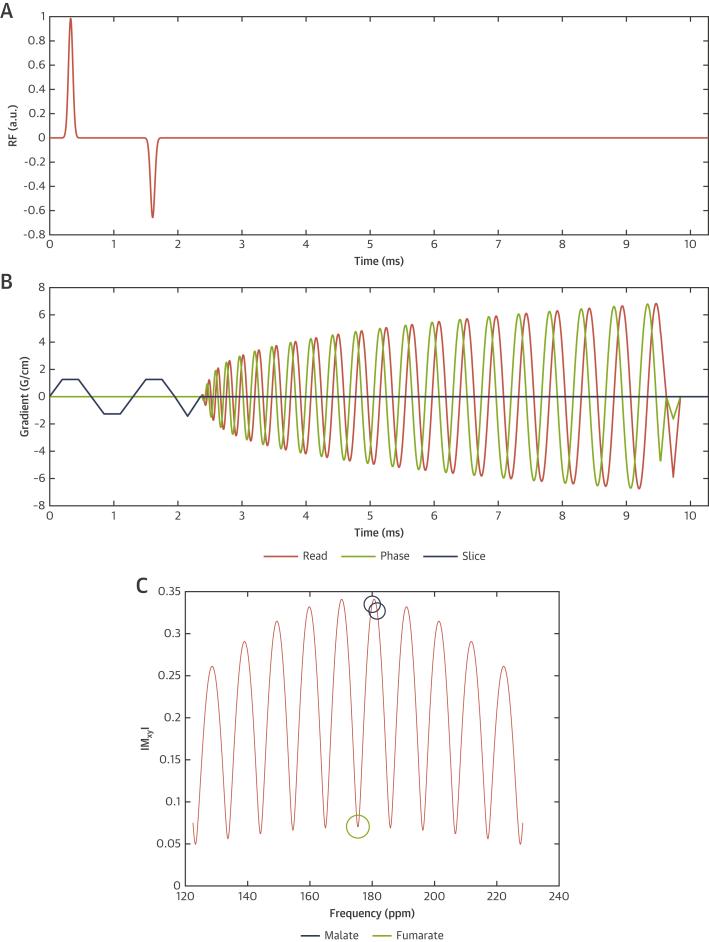
Magnetic Resonance Imaging Pulse Sequence Implemented to Achieve In Vivo Cardiac Malate Imaging **(A and B)** Multiband spectral-spatial excitation with spiral IDEAL multi-echo readout, showing both radiofrequency **(A)** and read/phase/slice gradient trajectories **(B)**. The corresponding frequency profile produced by the pulse **(C)** corresponds with a flip angle of ∼4° on the fumarate resonance, and approximately 20° on both malate peaks.

A novel minimum phase multiband spatial-spectral radiofrequency excitation pulse was designed to simultaneously excite [1,4-^13^C_2_]fumarate with a 4° flip angle, and both malate resonances with a 20° flip angle, with a chosen slice thickness of 20 mm. The pulse sequence developed is detailed in Figure 2 and the Online Appendix.

Proton images corresponding with the middle of the hyperpolarized imaging slice were acquired by CINE-MRI 29, 30. To quantify visually detected regions of akinesia, multidimensional feature tracking analysis was performed on CINE datasets using cmr42 (Circle, Calgary, Canada), and the long axis longitudinal and short axis circumferential myocardial strain calculated (31). Analyses of MR data are detailed in the Online Appendix.

### Statistical analysis

Statistical analyses were performed using GraphPad Prism. Perfused heart data were compared using the paired, nonparametric Friedman test, with the Dunn’s test implemented for post hoc multiple comparisons. Segmental linear regression was applied to investigate the relationship between energetics and malate production, fitting to slope values, the *x*-intercept, and the value *x*_0_ that signified the threshold for reduced ATP level that permitted cellular necrosis to proceed. Differences between groups in vivo were assessed by an unequal variance (Welch’s) *t* test with the Holm-Sidak post hoc correction for multiple comparisons. Statistical significance was considered at p < 0.05. Quoted statistics are mean ± SE of the mean.

## Results

### Cardiomyocyte fumarate uptake is negligible

To examine bulk fumarate uptake into the healthy myocardium we infused hyperpolarized [1,4-^13^C_2_]fumarate into the perfused heart and used magnetic resonance spectroscopy (MRS) to search for evidence of its enzymatic conversion into other species. Extremely low levels of metabolism were detected (Online Figure S1A). As an additional test, we measured mRNA expression of putative fumarate transporters in control heart tissue and tissue collected from hearts 45 min after reperfusion. Online Figure S1B shows that the genes encoding each transporter were detected robustly in kidney tissue (*Slc3a2* and *Slc3a3*, relative to internal control *18s*). The mRNA from *Slc3a2* could not be detected in heart tissue. Expression of *Slc3a3* was approximately 4 orders of magnitude lower than in the kidney, for both healthy and reperfused tissue (kidney, 4.1 × 10^−6^ ± 1.5 × 10^−6^; healthy heart, 4.2 × 10^−10^ ± 2.7 × 10^−10^; reperfused heart, 1.4 × 10^−9^ ± 3.7 × 10^−10^).

### Reperfusion compromises cell membrane integrity in the perfused heart

Hemodynamics resulting from our ischemia-reperfusion protocol are shown in the Online Appendix.

**Figure 3:**
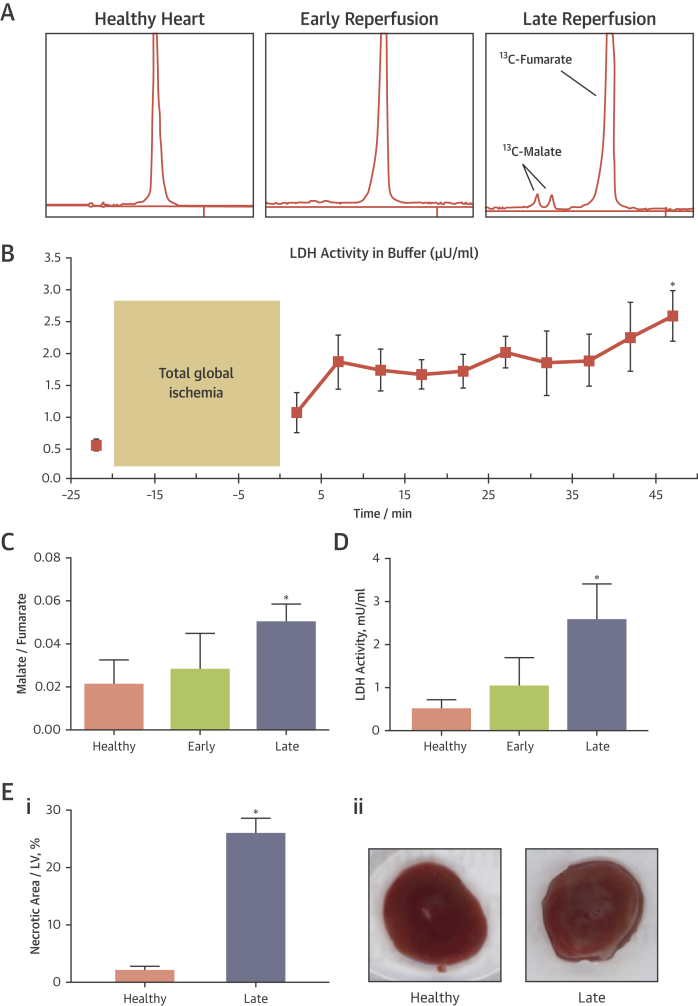
Comparison Between 2 Necrosis Measurements, ^13^C-Malate Spectroscopic Detection and LDH Release Into the Buffer, in Perfused Hearts **(A)** Representative MRS data from 1 perfused heart. **(B)** Average LDH release into the buffer throughout the experimental protocol, measured in 4 hearts. As in malate MRS, a trend toward increased buffer LDH activity was observed 2 min after reperfusion, but it did not reach significance until 45 min of reperfusion. **(C)** Quantification of ^13^C-malate detection across 6 hearts. **(D)** Quantification of LDH release at time points mirroring MRS data. **(E)** Necrotic region of the left ventricle (LV), expressed as a percentage, at the healthy time point, and after 2 h of reperfusion (when 2,3,5-triphhenyltetrazoliumchloride-staining can first reveal the necrotic region reliably 27, 28). As with malate MRS and LDH release, a significant increase in necrosis was observed after late reperfusion **(Ei)**. The degree of increase was larger (∼12×) due to ongoing necrosis during the reperfusion period (27). Representative triphhenyltetrazoliumchloride-stained heart slices are shown to the right **(Eii)**. ∗p < 0.05 compared with healthy group. Abbreviations as in Figure 1.

Upon infusion into the healthy heart, hyperpolarized [1,4-^13^C_2_]fumarate showed negligible conversion into [1,4-^13^C_2_]malate (Figure 3A). Immediately upon reperfusion, [1,4-^13^C_2_]malate was visible in some hearts, yet upon quantification the malate/fumarate ratio was not significantly increased (Figure 3C). Upon 45 min of reperfusion, a robust [1,4-^13^C_2_]malate signal was obvious in all hearts, and the malate/fumarate ratio increased 2.7-fold over baseline values.

MRS measurements of [1,4-^13^C_2_]malate production were compared with LDH release into the buffer and 2,3,5-triphhenyltetrazoliumchloride-staining for infarct area, historically gold standard markers for necrosis (32). In healthy hearts, buffer LDH activity was 0.71 ± 0.09 μU/ml. After 2 min of reperfusion, a time point that correlated with the MRS results, LDH activity was unchanged (1.0 ± 0.3 μU/ml). The LDH measurements during the reperfusion period showed a gradual upward trend in buffer LDH activity (Figure 3B). The LDH activity was increased significantly by 3.6-fold at the final time point (2.6 ± 0.4 μU/ml). In addition, 2,3,5-Triphhenyltetrazoliumchloride revealed that 2.2 ± 0.6% of the left ventricle of healthy isolated perfused hearts showed signs of necrosis. After ischemic injury and 2 h of reperfusion, 26.0 ± 2.5% of the left ventricle of the perfused hearts were necrotic, an increase of 11.8-fold.

### Necrosis correlates with myocardial energetics

**Figure 4:**
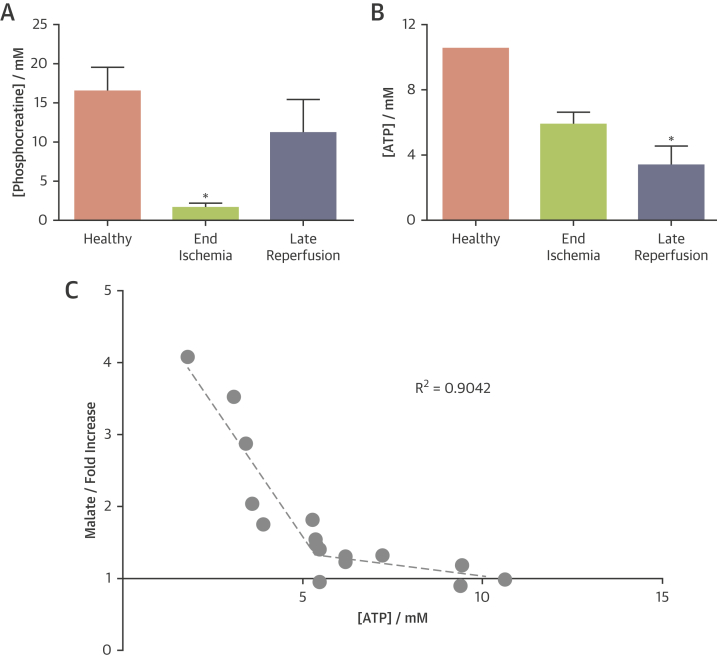
Quantification of High-Energy Phosphate Content of Perfused Hearts Throughout the Experimental Protocol Pseudoquantification of the [ATP] and [PCr] was performed by assuming that the [ATP] in baseline healthy hearts was 10.6 mmol/l in the fully relaxed 10-min acquisition. All spectra were referenced to this initial spectrum. Each parameter was measured from the spectra acquired over the final 5 min of the baseline period, of ischemia, and of reperfusion, respectively. **(A)** When averaged across the group of hearts, PCr was significantly reduced during ischemia and recovered after 45 min of reperfusion. **(B)** ATP was maintained throughout the ischemic period, but was depleted after 45 min of reperfusion. **(C)** Regression analysis revealed that [ATP] has a segmental relationship with ^13^C-malate production (expressed as malate/fumarate relative to the equivalent baseline value). When [ATP] was maintained above 5.3 mmol/l, very low levels of ^13^C-malate production were observed. Below this [ATP] threshold, each unit of [ATP] depletion (in mmol/l) resulted in a 74% increase in ^13^C-malate production. ∗p < 0.05 compared with healthy group. ATP = adenosine triphosphate; PCr = phosphocreatine.

In healthy hearts, the phosphocreatine (PCr)/ATP ratio was measured to be 1.6 ± 0.1, from which we calculated [PCr] = 16.5 ± 1.4 mmol/l. During ischemia, [PCr] was depleted to 1.59 ± 0.29 mmol/l, whereas [ATP] was not significantly reduced ([ATP] = 6.00 ± 0.29 mmol/l). After 45 min of reperfusion, [PCr] recovered to baseline values, yet [ATP] was depleted by 67% to 3.48 ± 0.46 mmol/l. Figure 4 summarizes these data.

Because ATP depletion has been implicated as an initiating event of necrosis, we examined the correlation between [ATP] (at all ^13^C examination points) and [1,4-^13^C_2_]malate production. The relationship (Figure 4C) showed a “thresholding” pattern, in which very little [1,4-^13^C_2_]malate production was observed when myocardial [ATP] was above 5.3 mmol/l (a 50% decrease), but when [ATP] dropped below this value, small changes to [ATP] enabled substantial changes to [1,4-^13^C_2_]malate. Segmental linear regression (R^2^ = 0.902) revealed that when myocardial [ATP] was maintained above 5.3 mmol/l, the slope was 0.065 mmol/l^−1^ (ATP depletion by 1 mmol/l caused a 6% increase in malate), whereas when [ATP] was below 5.3 mmol/l, each unit change in [ATP] resulted in a 74% increase in [1,4-^13^C_2_]malate.

### In vivo hyperpolarized ^13^C-fumarate MR

**Figure 5:**
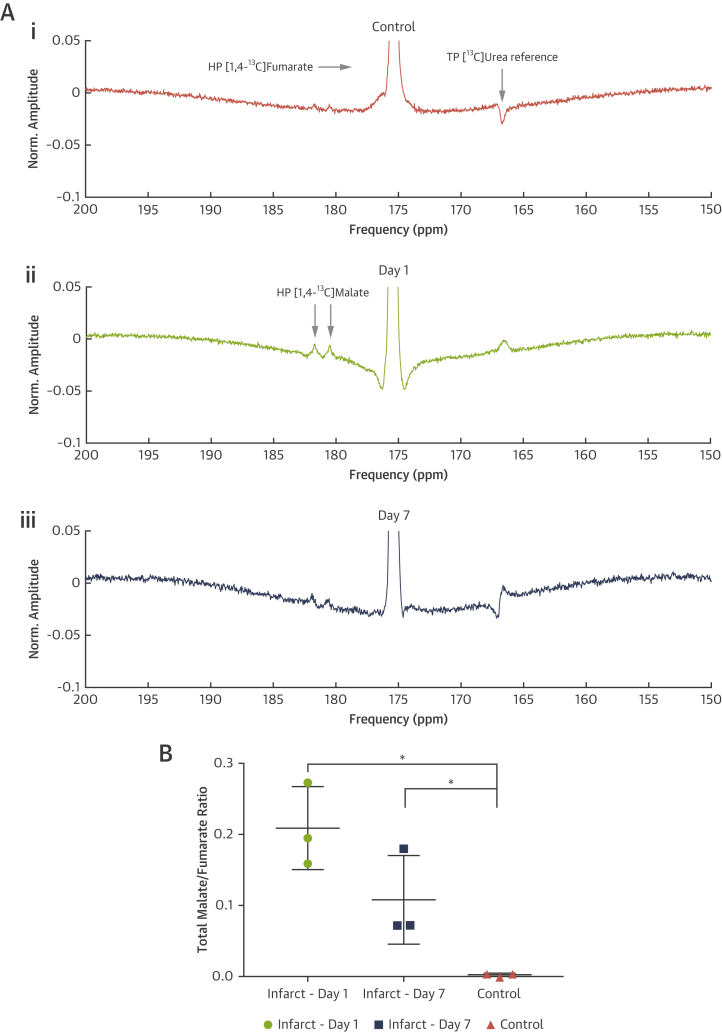
In Vivo ^13^C-Malate MR Detected the Acute Burst of Necrosis 24 h After Ischemia-Reperfusion Injury, as Well as After 1 Week **(A)** Example hyperpolarized [1,4-^13^C]fumarate spectra acquired from **(Ai)** control rats, or **(Aii)** 1 or **(Aiii)** 7 days post MI; spectra shown are temporally summed over 60 s after injection of fumarate. Spectra are shown normalized to the fumarate peak, and the quantity of visible [1,4-^13^C]malate produced is significantly different among all 3 groups **(B)**, with an approximately 82-fold increase in the total ([1-^13^C]+[4-^13^C])malate/fumarate 1 day post infarction compared with matched controls. For the 3 illustrative in vivo spectra shown, this ratio is 0.0035 (control), 0.1950 (day 1), and 0.0720 (day 7). ∗p < 0.05 compared with healthy group. HP = hyperpolarized; MI = myocardial infarction; MR = magnetic resonance; TP = thermally polarized.

**Figure 6:**
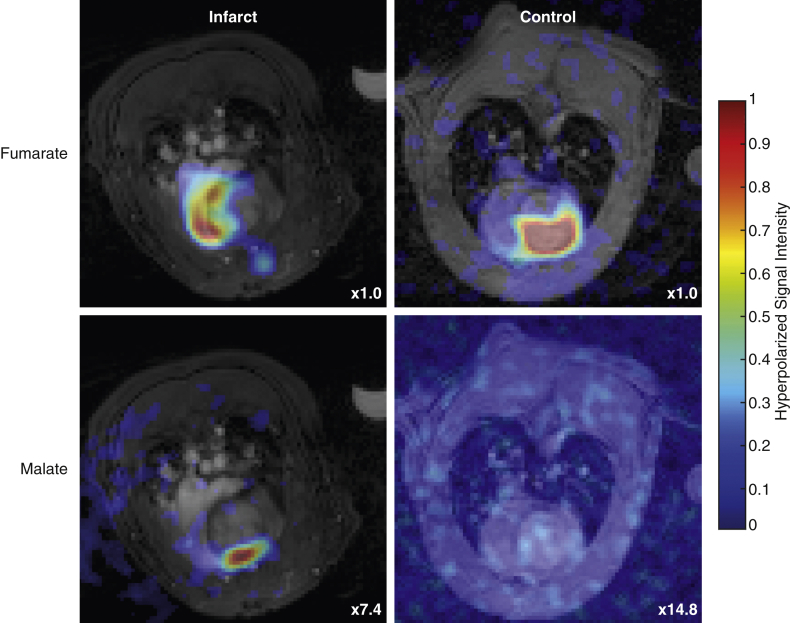
Spiral Multiband Pulse Sequence Developed Resolved Hyperpolarized [1,4-^13^C_2_]Fumarate Perfusing the Heart in Both Control and Infarcted Animals, 1 Day After Infarction [1,4-^13^C_2_]malate production was only visible in infarcted animals, and was localized to the anterior region of the chest wall, consistent with the location of the insult. The color axis is linear for the 4 hyperpolarized datasets, with the maximum level scaled down by the factor shown. Proton underlay images have been subject to a gamma correction ($\gamma =\frac{1}{2}$) to improve visualization of the cardiac structures.

**Figure 7:**
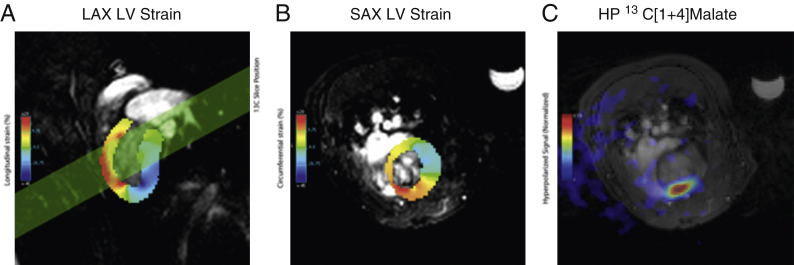
Peak Longitudinal and Circumferential Short Axis Myocardial Strain **(A)** Peak longitudinal long axis and **(B)** circumferential short axis myocardial strain 1 day after infarction as calculated via a feature-tracking algorithm and the acquired CINE datasets. The region of anomalous strain is consistent with the applied position of the cryo-probe on the proximal surface of the heart, and co-localizes with the region of visible hyperpolarized malate signal as obtained in the hyperpolarized experiment **(C)** depicted in Figure 6, together with its slice location as shown in **(A)**. HP = hyperpolarized; LAX = long axis; LV = left ventricle; SAX = short axis.

The quantity of [1,4-^13^C_2_]malate visible in the in vivo infarcted heart was significantly different among all 3 groups measured (Figure 5). The mean malate/fumarate ratio was 0.21 ± 0.03 1 day post infarction and decreased to 0.077 ± 0.005 7 days post infarction, compared with 0.0025 ± 0.001 in control animals. This striking ∼82-fold increase in visible malate signal post infarction corresponds to an estimated standardized effect size of approximately 5 (Cohen’s *d,* a scale-independent quantitative measure of effect strength, defined as the difference in means upon the SD) (33). The increased malate signal 1 day post infarction enabled the use of the spectral-spatial imaging sequence to spatially resolve the region of malate production (Figure 6). The region co-localized with an observed region of akinesia detected using CINE-MRI and quantified by computation of peak longitudinal and circumferential myocardial strain (Figure 7). It was not possible to resolve any regions of malate production in animals subject to sham surgery alone.

## Discussion

This study demonstrated that hyperpolarized [1,4-^13^C_2_]fumarate can be used with MR to identify regions of cardiomyocyte necrosis. Detection of necrosis was possible 1 day after MI, where an 82-fold increase in [1,4-^13^C_2_]malate production was observed, and up to 1 week later, when [1,4-^13^C_2_]malate production remained elevated by 31-fold over baseline. MR detection of the hyperpolarized [1,4-^13^C_2_]fumarate to [1,4-^13^C_2_]malate conversion offers the first method to noninvasively measure the rate and location of cardiomyocyte necrosis.

### Specificity to necrosis

The gold standard method for detecting cellular necrosis is by observing loss of membrane integrity via assays for uptake of dyes such as propidium iodide or the release of proteins such as troponin or LDH. Hyperpolarized malate MRI extends this concept into the realm of molecular imaging. The metabolic tracer hyperpolarized [1,4-^13^C_2_]fumarate is converted to [1,4-^13^C_2_]malate in the presence of fumarase only (21). In healthy tissue, fumarase is located intracellularly, making it inaccessible to intravenous [1,4-^13^C_2_]fumarate. However, with the early events of necrosis, fumarase is released into the extracellular space and can interact with infused [1,4-^13^C_2_]fumarate, to yield hyperpolarized [1,4-^13^C_2_]malate at a level that depends on the amount of released fumarase (21).

This study has generated evidence suggesting that, in cardiomyocytes, hyperpolarized [1,4-^13^C_2_]fumarate uptake and thus [1,4-^13^C_2_]malate production is near exclusively enabled by loss of membrane integrity. Our results confirmed that mRNA from the transporter encoded by *Slc3a3* was detected at a very low level 22, 23. Furthermore, given rapid ongoing rates of fumarate metabolism in cardiomyocytes, the lack of [1,4-^13^C_2_]malate production in healthy hearts strongly suggests negligible uptake.

### Necrosis, energetics, and contractile function in ischemic heart disease

During ischemia, acidosis reduces cardiac contractile function and minimizes ATP demand (34). During reperfusion, the heart’s contractile apparatus restarts and ion balance must be restored. In this study, ^31^P MRS measured preserved [ATP] immediately after ischemia alongside negligible levels of necrosis. After 45 min of reperfusion, ATP levels were reduced by 67% (below the threshold of 50% depletion suggested by our data), and histology, LDH release into perfusion buffer and malate MRS suggested increased necrosis. In general, the sudden elevation in ATP demand during reperfusion may be a fundamental contributor to reperfusion injury, exacerbating ATP depletion and ultimately permitting necrotic cell death (34).

Our data agree with numerous studies demonstrating a permissive role for ATP levels and irreversible myocardial damage 16, 17, 19, 35. However, the permissive value identified by our data was twice as high as that from previous work 19, 35. Additionally, our results diverge from previous work by suggesting a weak correlation between necrosis and ATP, and a stronger relationship between necrosis and lactate (19). The differences may be explained by differences in ATP demand, and the difference in parameters representing “necrosis” that were measured. Our study targeted a fundamental event of necrosis—cell membrane rupture—as an output, whereas previous work relied on scar size 19, 36. Scar may encompass other mechanisms of cell damage beyond necrosis that cloud the strength of the ATP–necrosis relationship (apoptosis, for example, requires ATP to proceed) (1). In cerebral infarction, experimental work has implicated tissue lactate as a predictor of destruction of the tissue at risk (37), whereas regions of ATP depletion pinpoint necrotic core (37). Future work with hyperpolarized [1-^13^C]pyruvate (to measure [1-^13^C]lactate production) alongside [1,4-^13^C_2_]fumarate and phosphorus-31 MRS may clarify if noninvasive metabolic biomarkers can provide similar predictive information to help guide therapy after MI.

The delay to cardiomyocyte necrosis resulting from MI lengthens the treatment window into the early reperfusion period for therapies that abrogate necrosis. Our results, in combination with the literature, suggest that necrotic cell death continues to increase up to 24 h after an MI. Pharmacological or mechanical blockade of programmed necrosis (e.g., by remote ischemic conditioning) at any point before this time could preserve myocardial viability 34, 38. Our data suggest that an approach to abrogating cardiomyocyte necrosis could involve maintaining [ATP] during reperfusion, by supporting its production or delaying the restoration of its use 39, 40. Further work is warranted to characterize energetic treatment strategies in the context of this treatment window.

### In vivo malate imaging

To interpret the results of a hyperpolarized [1,4-^13^C_2_]fumarate scan in the context of CVD, the implications of choosing an MRS or MRI acquisition must be understood. All values reported herein were quantified spectroscopically, averaging the ^13^C signal over the cardiac volume. This was optimal for the perfused heart, in which the mild, global insult caused diffuse cell death, as visualized by histology, and perfusion was maintained. However, MI causes localized cell death, leaving adjacent and remote tissue viable and potentially affecting regional perfusion. As such, the 82-fold increase in malate/fumarate averaged an intense focal region of cell death with regions of intact cardiomyocytes, and assumed that whole-heart perfusion was maintained. To observe necrosis within the damaged tissue volume, [1,4-^13^C_2_]malate MRI with high spatial resolution was required.

We developed and demonstrated a novel cardiac-gated sequence applied to free breathing animals in vivo, to sensitively detect spatially resolved malate production (and thus necrosis). The hybrid multiband spatial-spectral radiofrequency excitation pulse proposed forms a compromise between conventional chemical shift imaging MR sequences, which necessitate a long acquisition time, and the use of single-band spectral-spatial excitation pulses, which necessitate potentially unrealistically strong gradients for high-resolution imaging. In the context of hyperpolarized fumarate, the spectral-spatial approach is additionally challenging due to the close spectral separation of the malate doublet, which would necessitate a temporally long excitation pulse with a rapid imaging readout, leading to a comparative reduction in image domain signal to noise ratio (SNR). The sequence proposed, therefore, preserves longitudinal magnetization in the injected hyperpolarized fumarate without unduly sacrificing excitation time (and therefore SNR) while exciting any evolved malate present at a low concentration with a flip angle that is large enough for it to be detectable. The comparatively short duration of the signal-efficient readout trajectory used maximizes the SNR, and minimizes the effect of blurring due to motion. We note that the observed malate signal was primarily localized to the anterior wall of the myocardium, consistent with the central location of the infarct as inferred through feature tracking techniques. This finding is consistent with the hypothesis that the central region is highly necrotic, although further work will quantitatively correct for the nonuniform sensitivity profile of the receive array coil used in this study preventing undue weighting toward the chest wall, either via data-driven methods (if the data has sufficient SNR) or through the use of fiducial markers and electrodynamic calculations (in case of low SNR) (41). We expect future studies could quantitatively compare this region of malate with other MR markers of tissue injury, such as late gadolinium enhancement.

### Translational outlook

Hyperpolarized MR using [1-^13^C]pyruvate has been used in patients 24, 25. Translation of ^13^C-fumarate into the clinic has funding and is underway, implying that it will be the next tracer used clinically and making this study important for its imminent application in human CVD. The malate imaging sequence developed here is eminently translatable for future application in humans; scaling arguments predict that it would function with comparable anatomic resolution with greater SNR, and could be simplified to achieve malate imaging in other organs in which cardiac and respiratory motion do not present the same challenge. Spatially resolved hyperpolarized malate MRI could have a role in determining myocardial viability acutely in MI or to identify ongoing, low-grade cardiomyocyte drop-out that drives cardiac remodeling into heart failure that may not be detectable by bloodborne biomarkers. In addition, when applied in patients with chronic ischemia/angina, hyperpolarized malate MRI may prove valuable to evaluate the functional consequences of coronary artery disease or other vascular dysfunction and guide treatment decisions (42). Furthermore, malate imaging may be useful to identify and evaluate novel therapies that protect cardiomyocyte viability by abrogating cellular necrosis. Well-established approaches including rest and stress absolute myocardial blood flow with positron emission tomography or fractional flow reserve studies with computed tomography scans and coronary angiography are integrated in clinical practice for evaluation of epicardial coronary artery disease 15, 43, and hyperpolarized ^13^C-fumarate MRI may add to the physiological understanding of microvascular dysfunction. However, until ^13^C-fumarate MRI is investigated in human studies, its translational potential in this area remains speculative.

### Study limitations

Hyperpolarized ^13^C-fumarate will require several years of active development before it becomes available for patients. In the meantime, mechanistic work to characterize the relationships between necrosis, hyperpolarized ^13^C-fumarate delivery, fumarase clearance, and malate production in CVD would facilitate clinical translation. The MRI results presented here would be strengthened by correlation with histology. Future work should strive to identify the mechanism resulting in persistent malate production 7 days after MI; our results could be explained by the beginning of low-level cardiomyocyte necrosis that is known to occur during cardiac remodeling into failure (2), but could also indicate accumulation and persistence of released fumarase enzyme in the extracellular space. Understanding how long fumarase persists after MI will be essential in quantifying ^13^C-malate MRI signal as a marker of necrosis. Furthermore, correlating perfusion imaging techniques, such as first pass gadolinium or co-infusion with hyperpolarized ^13^C-urea (30), and ^13^C-fumarate imaging may enable the ^13^C-fumarate images themselves to facilitate interpretation of ^13^C-malate images in the context of altered perfusion. Ultimately, the application of ^13^C-fumarate imaging in patients with prior MI or coronary artery disease and comparison with current well-established imaging techniques will determine the value of this promising method.

## Conclusions

Malate production in the infarcted heart appears to provide a specific probe of necrosis acutely following MI, and for at least 1 week afterwards. This technique could offer a new noninvasive method to measure cellular necrosis in heart disease, and warrants further investigation in patients.

Perspectives **COMPETENCY IN MEDICAL KNOWLEDGE:** MR measurement of the hyperpolarized ^13^C-fumarate to ^13^C-malate conversion showed an 82-fold increase 24 h after an experimental MI (which persisted as a 31-fold increase after 1 week). This metabolic conversion could be imaged with a novel MRI pulse sequence, and correlated positively with other markers of necrosis: cell membrane integrity, wall motion abnormalities, and energetic depletion. Hyperpolarized ^13^C-fumarate may be used as a clinical MR tracer within several years, and could potentially offer the first method to noninvasively measure the rate and location of necrosis in CVD.

**TRANSLATIONAL OUTLOOK:** Pending development of the clinical hyperpolarized ^13^C-fumarate tracer, future basic science work should strive to characterize the relationships among necrosis, hyperpolarized ^13^C-fumarate delivery, fumarase clearance, and malate production. Furthermore, correlation with perfusion imaging techniques may facilitate interpretation of ^13^C-malate images in the context of altered perfusion.
