# Supplementary material for: Hyperpolarized [1,4-13C2]Fumarate Enables Magnetic Resonance-Based Imaging of Myocardial Necrosis
Source: JACC Cardiovasc Imaging. 2018 Nov;11(11):1594–606. doi: 10.1016/j.jcmg.2017.09.020 (PMC6231534; doi:10.1016/j.jcmg.2017.09.020)
Supplement: Online Data [file mmc1.docx]

**Supplemental methods**

**Contrast agent preparation:** [1,4-^13^C_2_]fumaric acid was prepared and hyperpolarized as previously described (1). Briefly, [1,4-^13^C_2_]fumaric acid (3.23 mmol; Cambridge Isotope Laboratories) was dissolved in 8.74 mmol of DMSO containing 11.48 µmol of a trityl radical (AH111501; GE Healthcare) and 0.48 µmol of a gadolinium chelate [Gd-3; GE Healthcare]. The solution was sonicated and centrifuged, and placed in the appropriate ^13^C polarizer system to polarize for 45 min.

For perfused heart experiments, a 10 mg aliquot of the [1,4-^13^C_2_]fumaric acid sample was hyperpolarized in a HyperSense polarizer (Oxford Instruments), and then dissolved in 3 ml of buffer containing 40 mM phosphate, 50 mM sodium chloride, and 20 mM sodium hydroxide, pH 7.4 at 10 bar and 180 °C. The resulting 3 ml of solution contained 10 mM [1,4-^13^C_2_]fumarate at 37 °C, which was rapidly mixed with 27 ml of perfusion buffer to yield a solution identical to the original perfusion buffer, supplemented with 1 mM hyperpolarized [1,4-^13^C_2_]fumarate, for subsequent MR spectroscopy experiments, as described below.

For *in vivo* experiments, a 40 mg aliquot of the [1,4-^13^C_2_]fumaric acid sample was polarized in an alpha-system polarizer (Oxford Instruments), and dissolved in 6 ml of buffer to yield a 20 mM solution of contrast agent. Two ml of hyperpolarized contrast agent (40 µmol fumarate) was infused into each rat for *in vivo* spectroscopy and imaging, as described below.

**Perfused heart experiments**

All investigations conformed to the Guide for the Care and Use of Laboratory Animals published by the US National Institutes of Health (NIH Publication No. 85-23, revised 1996), the Home Office Guidance on the Operation of the Animals (Scientific Procedures) Act, 1986 (HMSO), and to institutional guidelines. Male Wistar rats (∼300 g) were anaesthetized using a 0.7 mL i.p. injection of pentobarbital sodium (200 mg/mL Euthatal). Hearts were perfused in the Langendorff mode at a constant flow rate of 20 ml/min and 37°C temperature, as previously described (2). The Krebs–Henseleit bicarbonate perfusion buffer contained 11 mM glucose and was aerated with a mixture of 95% oxygen (O_2_) and 5% carbon dioxide (CO_2_) to give a final pH of 7.4 at 37°C.

Hyperpolarized [1,4-^13^C_2_]fumarate was infused into the healthy heart and the production of hyperpolarized [1,4-^13^C_2_]malate was followed using ^13^C MRS. This served as a control experiment. A fully relaxed ^31^P MR spectrum was acquired, followed by 10 min of dynamic ^31^P spectra. After the first ^31^P MRS spectra were acquired, hearts were placed into total global ischemia for 20 min, during which time ^31^P MRS acquisition was continued. Care was taken to ensure temperature was maintained at 37 °C. At the end of the ischemic time, flow was resumed with perfusion buffer containing hyperpolarized [1,4-^13^C_2_]fumarate, such that hyperpolarized [1,4-^13^C_2_]malate production could be monitored using ^13^C MRS within 30 s of initial reperfusion. ^31^P MRS spectral acquisitions were resumed within 5 min of initial reperfusion, for 40 min. After 45 min of reperfusion, hyperpolarized [1,4-^13^C_2_]fumarate was infused into the heart for a third and final time, and the production of hyperpolarized [1,4-^13^C_2_]malate was followed using ^13^C MRS. ^13^C and ^31^P MRS experiments were performed as previously described (2).

*Magnetic resonance spectroscopy (MRS):* ^31^P MR spectra were acquired at 202.5 MHz using a 30° radiofrequency (RF) pulse and a repetition delay of 0.25 s. The phosphocreatine (PCr) resonance was set at 0 ppm and the chemical shifts of all peaks were referenced to that of PCr. Each spectrum consisted of 120 transients, giving a total acquisition time of 30 s. As these partially saturated spectra had shorter repetition times than the longitudinal relaxation time of ^31^P nuclei, an unsaturated, ‘fully relaxed’ spectrum was initially acquired from the hearts using a 90° pulse with a repetition time of 15 s and 40 transients, and an acquisition time of 10 min. The unsaturated spectra were used to correct metabolite concentrations for the effects of saturation.

Acquisition of ^13^C MR spectra commenced immediately after infusion of hyperpolarized [1,4-^13^C_2_]fumarate and infusion continued throughout the acquisition. Spectra were acquired with 1 s temporal resolution over 2 min (excitation flip angle = 30°, 120 acquisitions). Spectra were centered at 150 ppm and 4096 points were acquired over a bandwidth of 100 ppm.

**Lactate dehydrogenase (LDH) activity assay**

Samples of perfusion buffer (1 ml) were acquired at baseline, after 2 min of reperfusion, and then at 5 min intervals throughout reperfusion, such that LDH in the perfusion buffer could be measured as a gold-standard marker of necrosis to compare with MRS. The LDH activity assay was performed according to the manufacturer’s instructions (Sigma Aldrich, Brøndby, Denmark) with few modifications. Tissue was homogenized in assay buffer specific for each activity kit, the solution was centrifuged, and the supernatant was stored at -80°C. The samples were distributed without pretreatment in a 96-well costar half plate. Analysis was performed in a PHERAstar FS micro plate reader (BMG Labtech, Birkerød, Denmark). Samples were stored in -80°C. The assays were performed using a full absorbance spectrum to precisely identify the absorbance peak: this was ±10 nm of the manufacturer’s given wavelength. The kinetic assay was performed over 45 min.

**RNA extraction and quantitative PCR**

Total RNA was isolated from myocardium using NucleoSpin RNA II mini kit according to the manufacturer’s instructions (AH diagnostics, Aarhus, Denmark). RNA was quantitated by spectrophotometry and stored at -80°C. cDNA synthesis was performed with RevertAid First strand cDNA synthesis kit (MBI Fermentas, Burlington, Canada). QPCR was performed using Brilliant SYBR Green qPCR Master Mix according to the manufacturer’s instructions (AH diagnostics, Aarhus, Denmark). Briefly, 100 ng of cDNA was used as template for PCR amplification. Specificity of products was confirmed by melting curve analysis and electrophoresis. Primer sequences used are given in table S1.

**Supplemental Table 1 –** *Primer sequences used for qPCR*

| **Gene** | **Forward** | **Reverse** |
| --- | --- | --- |
| *18s* | 5´-CAT GGC CGT TCT TAG TTG-3´ | 5´-CAT GCC AGA GTC TCG TTC-3´ |
| *Slc13a2* | AAGGCAGTGAGCAATCAGGT | TGAAGACAGATGGCTTGTGC |
| *Slc13a3* | CCCTCACTCAAGTGGTGGTT | GGCGAACTCTGTGAAGAAGG |

**Quantification of infarct area**

Area of infarct due to necrosis was quantified using triphenyltetrazolium chloride (TTC) staining, a colorless dye that is reduced to a deep-red precipitate by dehydrogenases in the presence of NADH. TTC stain must only be performed after dehydrogenase washout is complete; 2 hours for crystalloid-perfused Langendorff hearts is considered to be the minimum time acceptable (3,4).

As such, additional cohorts of hearts was perfused according to the protocol above, but either frozen after the 20 min stabilization period (n=3) or reperfused for 2 hours (n=4), to best mimic the healthy and late time points of the MR protocol. At the end of the protocol hearts were removed from the perfusion apparatus, frozen at − 80 °C for 20 min and subsequently sliced into ~ 1.5 mm thick transverse sections. Slices were submerged in a TTC solution (1% in phosphate buffer, pH = 7.4 and 37 °C) for 3 min rendering vital tissue deep red and leaving infarcted/necrotic tissue pale. Hearts were stored in 4% formaldehyde (Lillies Solution, VWR-Bie & Berntsen, Herlev, Denmark) for 24 h to enhance contrast between vital and infarcted tissue. Each slice was weighed and scanned on a flatbed scanner (HP ScanJet 4300C, Hewlett Packard, Palo Alto, CA, USA). Area-at-risk (corresponding to the left ventricle, LV) and infarct size were measured by manual delineation (ImageJ, NIH) enabling calculation of an infarct size/area-at-risk ratio weighted with the mass of each individual slice.

**MRS Data analysis**

#### *Carbon-13 MRS from the perfused heart:* Cardiac ^13^C MR spectra were analyzed using the AMARES algorithm, as implemented in the jMRUI software package (5). Sixty seconds of spectra were summed, DC offset corrected based on the last half of acquired points and peaks corresponding with [1,4-^13^C_2_]fumarate and its metabolic derivatives were fitted after liner phase correction assuming a Lorentzian line shape, initial peak frequencies, relative phases, and linewidths. The asymmetrical [1,4-^13^C_2_]malate doublet peak areas were summed together and data were expressed as an overall malate:fumarate ratio.

#### *Phosphorus-31 from the perfused heart:* Cardiac ^31^P MR spectra were analyzed using the AMARES algorithm in the jMRUI software package, as described previously (2). Briefly, spectra were corrected for DC offset using the last half of acquired points. The PCr, P_i_, α-, β-, and γ-ATP resonances were fitted assuming a Lorentzian line shape, peak frequencies, relative phases, linewidths, and J-coupling parameters. pH_i_ was calculated from the P_i_ chemical shift. Absolute ^31^P metabolite concentrations were calculated using an ATP concentration of 10.6 mM from the first γ-ATP peak area and expressing all other ATP peak areas relative to this area.

**Myocardial infarction surgery**

Six female Wistar rats (mass ≈ 200 g, Harlan, UK) were divided into two groups (MI or control, n=3 each). Rats were anaesthetised via isoflurane in oxygen (4% for induction, 2% for maintenance) and intubated for ventilation (~250 µl/min, 70 breaths/min; Harvard Apparatus). Rats were maintained on a heated pad with monitoring of temperature, pulse oxygenation and ECG (MouseMonitor S, Indus Instruments).  Following a left thoracotomy and removal of the pericardium, the heart was stabilised by a loose stitch through the apex and myocardial infarction was induced by cryoinjury, via the manual attachment of a 10 mm ø aluminium cylindrical probe initially at 77 K onto the ventral apical epicardial surface of the LV for 15 seconds. The chest was closed and the animal allowed to recover. In control animals, the chest was closed after removal of the pericardium. Animals were provided with pre-operative and postoperative analgesia, in the form of buprenorphine, metacam and topical local anaesthesia with lidocaine, and were monitored regularly following the procedure.

**In vivo MR**

Rats were anaesthetized with isoflurane in oxygen cannulated via the tail vein, and imaged using a Varian (Santa Clara, California, USA) 7 T DDR system. Physiological monitoring and homoeothermic maintenance was provided using a custom-built handling system that ensured temperature regulation and provided RF shielded ECG/RF coil interfaces (6).

*Carbon-13 malate imaging:* In order to spatially resolve the expected small amplitude of the signal coming from any evolved malate, a novel minimum phase multiband spatial-spectral RF excitation pulse was designed to simultaneously excite [1,4-^13^C_2_]fumarate with a 4º flip angle, and both malate resonances with a 20º flip angle, with a chosen slice thickness of 20 mm (minimum achievable slice thickness was 5.74 mm at 175 mT/m and 875 mT/m/ms). The multiband pulse consisted of a gauss-gauss tip-up tip-down scheme, chosen to provide the opportunity to both avoid exciting [1,4-^13^C_2_]fumarate with an excessively large flip angle without also requiring a substantially longer single-band spectral-spatial excitation pulse that may otherwise cause excessive T_2_^*^-mediated signal loss. A multi-echo spiral “IDEAL” readout maximised the effective number of signal averages over all chemical species in the subsequent IDEAL reconstruction, with an FID acquisition occurring every seventh echo to guide the subsequent reconstruction (TE=2.1, 3.8, 5.5, 7.2, 8.9, 10.6, 12.3, FID 2.1 ms) (7). The golden-angle spiral trajectory was designed as described previously (8), with a nominal FOV of 80×80 mm^2^, a readout bandwidth of 250 kHz, TR=1 RR interval, nominal acquisition in-plane resolution 2×2 mm^2^. The pulse sequence used is shown in Figure 1. The temporal resolution of the imaging sequence was 8 heartbeats (~1.2 s), and the images presented are those summed for approximately 30 s following the appearance of the fumarate bolus. A ^13^C-urea phantom was included to perform an appropriate power calibration prior to each experiment, and used as a frequency reference. In order to ensure adequate B_0_ homogeneity to mitigate the sensitivity of both the spiral readout and multiband excitation to errors in transmitter frequency, a (proton) cardiac gated multi-echo impulse-response based automated shimming procedure was run prior to each experiment . Proton images corresponding to the middle of the hyperpolarised imaging slice were acquired by CINE (8,9).

***In vivo* MR data analysis**

*Carbon-13 MRS:* Spectral multi-coil recombination was performed by computing the noise decorrelation matrix according to the method of Kellman and McVeigh (10), followed by the separate phasing and addition of each spectrum. Spectra were subsequently summed for 60 s following the appearance of the [1,4-^13^C_2_]fumarate peak, and further quantified with AMARES as for the perfused heart, with the reported malate:fumarate ratio being that of total visible malate (i.e. [1-^13^C]+[4-^13^C]malate peak amplitudes summed over time) to total (temporally summed) fumarate.

*Carbon-13 malate MRI:* In order to ameliorate the blurring effect of gradient infidelities, a previously measured gradient impulse response function (GIRF, c.f. (11)) was used to predict the spiral trajectory experimentally taken from that prescribed. The IDEAL reconstruction performed by computing the Moore-Penrose pseudo-inverse of the Fourier encoding matrix $A_{m,n}=e^{-i \text{TE }_{n} \omega_{m}}$ and then applying it to each spatial *k*-space point in the multi-echo dataset (12). This reconstruction tacitly assumes B_0_ homogeneity in the heart, and the interleaved FID acquisition allows both for the centre (fumarate) frequency to be set accurately and an assessment of the homogeneity criterion made. The acquired samples were then transformed via non-uniform FFT after density compensation and exponential filtering in the time domain by 30 Hz (13). The reconstructed in-plane resolution was 5.62 $\times$ 5.62 mm^2^, determined by explicit calculation of the point spread function, with a nominal reconstructed matrix size of 128$\times$128. After reconstruction, the multi-coil data were whitened and recombined as described above, minimising the imaginary part of the dataset, and images corresponding to both malate peaks were combined in quadrature.

#### Supplemental results

***Function of the isolated perfused heart***

Initially upon perfusion, healthy hearts developed left ventricular (LV) pressure of 75.2 ± 4.2 mmHg and beat with a heart rate of 297 ± 22 bpm, yielding a rate-pressure product (RPP) of 22097 ± 1620 mmHg$\cdot$bpm. During ischemia, contractile function rapidly ceased and after approximately 10 min LV end diastolic pressure increased by 210% to 41.2 ± 4.4 mmHg. After 45 min of reperfusion, heart rate recovered to the initial value but LV developed pressure was reduced by half (to 35.8 ± 3.5 mmHg) and LV end diastolic pressure remained elevated. RPP in the reperfused heart was 10029 ± 1664 mmHg$\cdot$bpm.

***Quality assurance for* in vivo *13C MRS and MRI data***

The quality of the shim was verified via the acquisition of 3D multi-echo cardiac gated B_0_ maps for all experiments. Three example field maps are shown in Figure S3A, corresponding with control, Day 1, and Day 7 time points from a single rat. Note that all three images are approximately homogeneous over the heart, despite variation in the centre frequency of the transmitter (and hence bulk ‘color’ and the color axis scale of each image). Most inhomogeneity appears to arise due to ghosting from residual respiratory motion or be nearer the subcutaneous fat.

A decrease in B_0_ homogeneity would result in an increased linewidth for each metabolite in the heart, which could in turn interfere with our ability to robustly detect the malate signal, particularly in the control condition. As such we assessed the linewidths of each peak for each animal and time point examined. No statistically significant difference in linewidths between the groups was observed. Furthermore the linewidth for the urea phantom was consistent between experiments, and although the phase of the peak varies between absorption and dispersion, it was always well approximated by a Lorentzian shape, as expected *a priori* for a well-shimmed liquid sample. A summary graphical representation of the linewidths for each metabolite is shown in Figure S3B.

**Supplemental figures**

**Figure S1** Fumarate uptake into the healthy myocardium occurs at a very low rate. **A)** In healthy perfused hearts, infusion with 1 mM hyperpolarized [1,4-^13^C_2_]fumarate and subsequent MRS detection resulted in minimal [1,4-^13^C_2_]fumarate to malate conversion. The spectrum on the right shows the same information as the spectrum on the left, but with the vertical scale reduced by 10-fold. For both sets of spectra, 2 min of acquisition with a 30 degree flip angle were summed. No line broadening was applied. b) Quantitative, real-time PCR analysis of the expression of the *Slc3a2* and *Slc3a3* genes, which encode the putative dicarboxylic acid transporters that mediate fumarate cellular uptake. Kidney tissue was used as a positive control, and in all tissue, 18s was used as an internal reference. *Slc3a2* was not detected in heart. *Slc3a3* was barely detectable in heart, at a level 4 orders of magnitude lower than in kidney.

**Figure S2** The function effects of the ischemia-reperfusion protocol. **A)** Representative raw data from a single heart showing the evolution of developed pressure, from the point at which hearts were perfused, through the baseline and ischemic periods, and during 45 min of reperfusion. **B, C, D,** and **E)** Respectively, the quantifications of developed pressure, heart rate, end diastolic pressure and rate pressure product averaged from all hearts. Each parameter was measured from the raw pressure traces over the final 5 min of the baseline period, the final 5 min of ischemia, and the final 5 min of reperfusion.

#### Figure S3 Analyses performed to assess the quality of *in vivo* MRS and MRI data acquisition. A, top) Three representative B_0 f_ield maps, corresponding with control, Day 1, and Day 7 time points from a single rat. Field maps were acquired using a 3D multi-echo cardiac gated sequence for every *in vivo* experiment performed, and served as an initial evaluation to ensure adequate B_0_ homogeneity following application of the a cardiac gated auto-shim algorithm. B, bottom) A summary graphical representation of the linewidths for each metabolite, alongside that of the urea phantom.

**Figure S1**

**Figure S2**

**Figure S3**

**References**

1. Gallagher FA, Kettunen MI, Hu DE et al. Production of hyperpolarized [1,4-13C2]malate from [1,4-13C2]fumarate is a marker of cell necrosis and treatment response in tumors. Proc Natl Acad Sci U S A 2009;106:19801-6.

2. Schroeder MA, Swietach P, Atherton HJ et al. Measuring intracellular pH in the heart using hyperpolarized carbon dioxide and bicarbonate: a 13C and 31P magnetic resonance spectroscopy study. Cardiovascular research 2010;86:82-91.

3. Povlsen JA, Lofgren B, Dalgas C, Jespersen NR, Johnsen J, Botker HE. Frequent biomarker analysis in the isolated perfused heart reveals two distinct phases of reperfusion injury. Int J Cardiol 2014;171:9-14.

4. Jespersen NR, Yokota T, Stottrup NB et al. Pre-ischaemic mitochondrial substrate constraint by inhibition of malate-aspartate shuttle preserves mitochondrial function after ischaemia-reperfusion. J Physiol 2017;595:3765-3780.

5. Naressi A, Couturier C, Castang I, de Beer R, Graveron-Demilly D. Java-based graphical user interface for MRUI, a software package for quantitation of in vivo/medical magnetic resonance spectroscopy signals. Comput Biol Med 2001;31:269-86.

6. Schneider JE, Cassidy PJ, Lygate C et al. Fast, high-resolution in vivo cine magnetic resonance imaging in normal and failing mouse hearts on a vertical 11.7 T system. J Magn Reson Imaging 2003;18:691-701.

7. Wiesinger F, Weidl E, Menzel MI et al. IDEAL spiral CSI for dynamic metabolic MR imaging of hyperpolarized [1-13C]pyruvate. Magn Reson Med 2012;68:8-16.

8. Lau AZ, Miller JJ, Robson MD, Tyler DJ. Simultaneous assessment of cardiac metabolism and perfusion using copolarized [1-13 C]pyruvate and 13 C-urea. Magn Reson Med 2016.

9. Miller JJ, Lau AZ, Teh I et al. Robust and high resolution hyperpolarized metabolic imaging of the rat heart at 7 T with 3D spectral-spatial EPI. Magn Reson Med 2016;75:1515-24.

10. Kellman P, McVeigh ER. Image reconstruction in SNR units: a general method for SNR measurement. Magn Reson Med 2005;54:1439-47.

11. Duyn JH, Yang Y, Frank JA, van der Veen JW. Simple correction method for k-space trajectory deviations in MRI. J Magn Reson 1998;132:150-3.

12. Reeder SB, Wen Z, Yu H et al. Multicoil Dixon chemical species separation with an iterative least-squares estimation method. Magn Reson Med 2004;51:35-45.

13. Zwart NR, Johnson KO, Pipe JG. Efficient sample density estimation by combining gridding and an optimized kernel. Magn Reson Med 2012;67:701-10.
